# Aminoglycoside Stress Together with the 12S rRNA 1494C>T Mutation Leads to Mitophagy

**DOI:** 10.1371/journal.pone.0114650

**Published:** 2014-12-04

**Authors:** Jialing Yu, Jing Zheng, Xiaoxu Zhao, Junxia Liu, Zhuochao Mao, Yining Ling, Danni Chen, Chao Chen, Lanlan Hui, Limei Cui, Ye Chen, Pingping Jiang, Min-Xin Guan

**Affiliations:** 1 Institute of Genetics, Zhejiang University, Hangzhou, China; 2 Collaborative Innovation Center for Diagnosis and Treatment of Infectious Diseases, Zhejiang University, Hangzhou, China; 3 School of Basic Medical Sciences, Zhejiang University, Hangzhou, China; 4 School of Agriculture and Biotechonlogy, Zhejiang University, Hangzhou, China; Newcastle University, United Kingdom

## Abstract

Aminoglycosides as modifying factors modulated the phenotypic manifestation of mitochondrial rRNA mutations and the incomplete penetrance of hearing loss. In this report, using cybrids harboring the m.1494C>T mutation, we showed that gentamycin aggravated mitochondrial dysfunction in a combination of the m.1494C>T mutation. The m.1494C>T mutation was responsible for the dramatic reduction in three mtDNA-encoded proteins of H-strand, with the average of 39% reduction, except of the MT-ND6 protein, accompanied with 21% reduction of ATP production and increase in mitochondrial reactive oxygen species, compared with those of control cybrids. After exposure to gentamycin, 35% reduction of mitochondrial ATP production was observed in mutant cybrids with a marked decrease of the mitochondrial membrane potential. More excessive cellular reactive oxygen species was detected with stimulus of gentamycin than those in mutant cells. Under gentamycin and m.1494C>T stress together, more dysfunctional mitochondria were forced to fuse and exhibited mitophagy via up-regulated LC3-B, as a compensatory protective response to try to optimize mitochondrial function, rather than undergo apoptosis. These findings may provide valuable information to further understand of mechanistic link between mitochondrial rRNA mutation, toxicity of AGs and hearing loss.

## Introduction

Since the m.1555A>G and m.1494C>T mutations were first reported in families with aminoglycoside-induced and nonsyndromic hearing loss (AINHL) [1], [2], aminoglycosides (AGs) have been identified as one kind of modifying factors for hearing loss, modulating the phenotypic manifestation of m.1555A>G or m.1494C>T mutations. The well established molecular mechanism is that m.1555A>G or m.1494C>T mutation creates a G-C or A-U base pairing at the A-site of mitochondrial ribosome which could make the human mitochondrial ribosomes more bacteria like and more susceptible to AG binding [3]–[6]. Some observations have shown that the presence of m.1555A>G or m.1494C>T mutation does increase the drug susceptibility [7], [8]. Thus, AGs are usually assumed to impair mitochondrial translation in susceptible subjects carrying either of these two deleterious mutations. Guan, *et al.* (2000) [9] reported that following treatment with paromomycin, a reduction of 30% or 28% in mitochondrial protein synthesis was observed in lymphoblastoid cell lines carrying the A1555G mutation derived from symptomatic or asymptomatic individuals, respectively. However, it still hard to distinguish the reduction of mitochondrial protein synthesis is directly due to single-gene mutation or AG causes or a combination of the two. Additionally, since mitochondria are strongly implied as a primary targets in hearing loss induced by AGs [10], [11], more and more evidences have demonstrated that AGs not only decrease mitochondrial ATP synthesis [12], but also induce excessive reactive oxygen species (ROS) production, which may trigger multiple forms of cell death via the JNK/MAPK or BCL2 pathway [13], [14]. It seems that AGs have wide-spectrum effects on mitochondrial function besides their effect on mitochondrial translation. As a consequence, AGs are reported to worsen hearing loss in individuals carrying these two mutations [15], [16]. But the specific mechanistic link between mitochondrial rRNA mutation, AG ototoxicity and hearing loss remains largely elusive. Here we hypothesize that AGs may enhance the effect of these two mutations on mitochondrial function, and especially underscore how mitochondria respond to stresses due to both AG toxicity and genotoxicity *in* *vitro.* Although most research has focused on apoptosis that have been documented for gene mutation and/or AGs in hearing loss [17], [18], a few observations have proposed that mitochondria may elicit compensatory protection to rescue the cell from death on stresses [19]. Morphological alteration of mitochondria has been observed after exposure to kanamycin in hair cells of guinea pigs [20]. Likewise, mitochondrial fusion or mitophagy can be observed in dysfunctional mitochondrial population to maintenance of bioenergetic capacity under stresses or disorders [21], [22]. Therefore we also assume that compensatory protective activities may occur as initial effects of AG toxicity with genotoxicity together on mitochondrion.

In our previous study, the m.1494C>T mutation was associated with AINHL in a large Han Chinese pedigree [15]. It documented that the defect of mitochondrial protein labeling was contributed to the m.1494C>T mutation. In order to confirm our hypothesis, three cybrid cell lines derived from symptomatic members in this family and three cybrids from genetically unrelated Chinese control subjects are used in this study. Here we show how gentamycin worsen the defect of mitochondrion associated with the m.1494C>T mutation. After exposure to gentamycin, efficiency of ATP production is decreased in cybrids carrying the m.1494C>T mutation. The reduced mitochondrial membrane potential and mitochondrial ROS formation in mutant cybrids are subjected to gentamycin exposure. Under conditions of drug stress with the mutation together, more population of dysfunctional mitochondria is forced to fuse and displays mitophagy rather than apoptosis.

## Materials and Methods

### Cybrid Cell Lines and Culture Conditions

Six cybrid cell lines derived from three affected matrilineal relative (III-12, III-18 and IV-21) and three genetically unrelated Chinese control subjects (A3, A6 and A7) [2], [15] were named as 1494T1, 1494T2, 1494T3, A3t, A6t and A7t respectively. All cybrids were constructed by transferring mitochondria from immortalized lymphoblastoid cell lines into human mtDNA-less cells (ρ°206) [23]. As ρ°206 cell line was derived from the bromodeoxyuridine (BrdU) resistant 143B.TK^−^ (143B) cell line, 143B was also used as the parental line in our assessment, grown in DMEM with 100 µg of BrdU per ml, 1 mM pyruvate and 5% FBS according to published protocols [24]. Cybrids used here were maintained in the same medium as the 143B.TK^−^ cell line. Additionally, mtDNA genome of cybrids was carried out to confirm that it was same as to its lymphoblastoid cells. All selected cybrids had similar mtDNA copy numbers as shown in Figure S1, performed as described elsewhere [25]. When the m.1494C>T cybrid explored to gentamycin, we named it as double-hit cybrid.

### Western Blotting

Cybrids, either subjected to gentamycin treatment or not, were homogenized in ice-cold RIPA lysis buffer. The protein concentration was quantified using the Bradford Protein Assay Kit (Bio-Rad Laboratories, Inc.), and 20 µg of each protein was separated on 12% SDS-polyacrylamide gels. The primary antibodies used for the synthesis of mitochondrial polypeptides were anti-ND5, CO1, CO2 (Abcam) and ND6 (Santa Cruze). An antibody against the lipid-conjugated MAP1LC3 (LC3-B) protein (Cell Signaling Technology) was prepared for the analysis of mitophagy at a 1/1,000 dilution. Anti-tubulin (AT819, Beyotime) served as a loading control. The resultant western blots were scanned with Clinix and analyzed using Carestream MI SE software. The band densities were normalized to the background. The probing protein/tubulin ratio was then calculated from the band densities. Finally, the statistical significance of the difference in the ratio of the control to the experimental bands was determined.

### ATP Measurement

A total of 10,000 cells from each well were seeded in white opaque tissue culture plates and then exposed, with or without 2 mg/ml of gentamycin, for 24 hours before measurement. The measurements of cellular and mitochondrial ATP production were performed following procedures elsewhere [26]. Briefly, the cells were incubated for 2 hours with 10 mM glucose (total ATP production) or 5 mM 2-deoxy-D-glucose (2-DG) plus 5 mM pyruvate (for mitochondrial ATP production). According to the manufacturer’s instructions, 1 volume of buffer plus substrate (CellTiter-Glo® Luminescent Cell Viability Assay kit, Promega), was added to the plate to cause the cells to lyse completely, and followed by incubation for 10 min at room temperature and measurement with Synergy H1. The rate of ATP efficiency was determined as the production of mitochondrial ATP *vs.* total cellular ATP and normalized to the average control value.

### ROS Measurements

Approximately 2×10^5^ cells, with or without 2 mg/ml gentamycin loading, were incubated for 24 hours before being harvested. Total cellular ROS generation was detected with 20 µM 2′, 7′-dichlorodihydrofluorescein diacetate (ab113851, Abcam) using an FC500 MCL (Beckman Coulter), with an excitation wavelength of 488 nm and emissions wavelengths of 530 nm.

ROS generation by mitochondria in living cells was analyzed using the mitochondrial superoxide indicator MitoSOX-Red (Invitrogen). The same density of cells was seeded into 6-well plates, with or without 2 mg/ml of gentamycin, followed by incubation for 24 hours. According to the manufacturer’s instructions, 5 µM MitoSOX was added to the plates, followed by incubation for 10 mins at 37°C. The cells were then washed twice with pre-warmed HBSS Ca/Mg and detached with 0.25% trypsin. Fluorescence was measured using a FACSCalibur (BD Biosciences), with excitation at 488 nm and emission at 580 nm. The data were analyzed with FlowJo software.

### Analysis of the Mitochondrial Membrane Potential

A total of 10,000 cells were seeded in each well of black opaque cell plates and treated with either 2 mg/ml of gentamycin or PBS as a control for 24 hours. Then, following the manufacturer’s instructions, treatment with the JC-10 kit (Abcam, ab112134) was performed as follows: 50 µl of JC-10 was added per well, followed by incubation for 1 hour before adding buffer B. Fluorescence was subsequently recorded using a Synergy H1 (BioTek) at an excitation wavelength of 488 nm and emission wavelengths of 529 nm and 590 nm. The 590 nm *vs*. 529 nm ratio served as the relative membrane potential.

### Quantitative RT-PCR

Total RNA was isolated from each of the cell lines using the RNeasy plus Mini kit (Qiagen). The quality and quantity of the obtained RNA were determined using a Nano Drop (Thermo Scientific). The RNA (1 µg) was then reverse-transcribed to first-strand cDNA using SuperScript III reverse transcriptase. The cDNA was then diluted 1∶10 with DEPC-H_2_O, and real-time PCR was performed in a 7900 HT Fast Real-time PCR System (Applied Biosystems) using SYBR Green Master Mix (Roche Applied Science). Triplicate reactions were run for each sample in 96-well plates with the first-strand cDNA as the template. Relative gene expression was analyzed using the 2^−ΔΔ*CT*^ method via normalization to *18S* rRNA levels, as described elsewhere [25]. All of the primers used in this study are listed in Table S1.

### Immunocytochemistry Analysis of Mitochondrial Morphology

Cybrids either treated with gentamycin or not were cultured on collagen-coated cover glass slips (Thermo Fisher) for 24 hours in the presence of serum. To visualize the mitochondrial network, tetramethylrhodamine methyl ester (TMRM) fluorescence (Invitrogen) was employed. The cells grown on coverslips were incubated in growth medium supplemented with 100 nM TMRM for 30 min, then washed in warm fresh medium, mounted, and visualized using an Olympus Fluoview FV1000 (Olympus, Japan) with a 60× objective.

### Cell Apoptosis Assay

Apoptosis in the cybrids treated with or without gentamycin was determined using the Annexin V-FITC Apoptosis Kit (BioLegend, 640914) according to manufacturer’s protocol. A total of 2×10^5^ cells/ml were seeded in 6-well culture plates and treated with or without gentamycin for 24 hours. Then, all of the harvested cells were stained with Annexin V and PI for 15 min at room temperature and subsequently analyzed via flow cytometry (FACS Calibur, BD Biosciences). The data were analyzed using FlowJo software.

### Statistics

The data are presented as the Mean±Standard deviation. Student’s unpaired, two-tailed t-test was used to compare the obtained values, and a histogram was constructed using GraphPad Software. A *P* value of<0.05 was considered statistically significant.

## Results

### Reduction in the Level of Mitochondrial Proteins Due to the m.1494C>T

**Figure 1:**
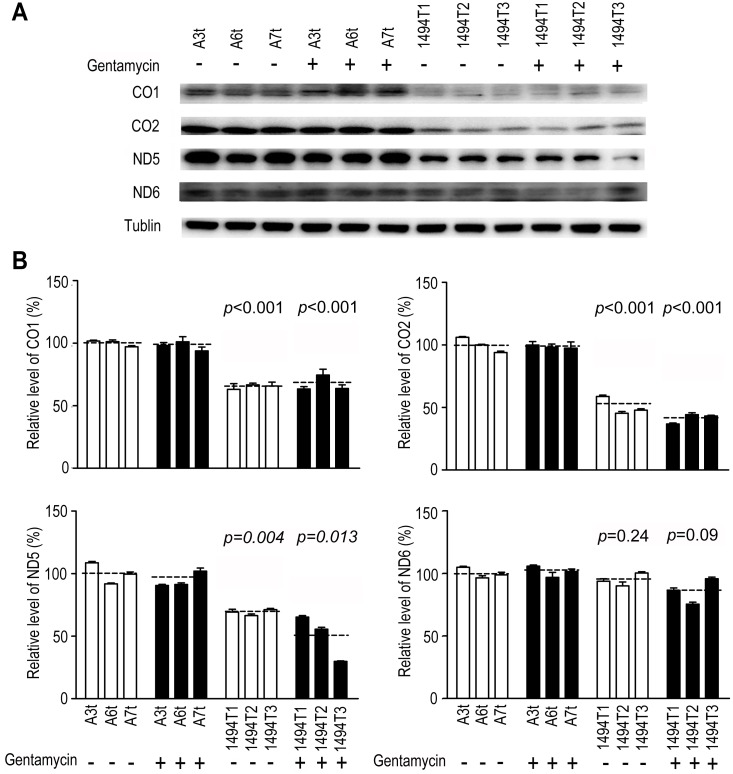
Western blot analysis of mitochondrial protein. (A) Samples of 20 µg of total protein from the whole-cell lysates of the cybrids without (−) or with (+) gentamycin stimulation were electrophoresed and subjected to blotting with four respiratory complex subunit antibody: anti-ND5, CO1, CO2 (Abcam) and ND6 (Santa Cruze). Tubulin served as a loading control. (B) Quantification of the levels of each protein subunit. The average relative contents per cell were normalized to the average content of tubulin per cell. The final normalization data were obtained from the mean value in the control cells without gentamycin stimulation as relative percentages. The data represent the mean ± s.d. of three independent experiments; Student’s *t*-test, unpaired, two-tailed.

Our previous observations revealed that the ratios of the translation rates of mitochondria in cell lines carrying m.1494C>T was significantly decreased compared with the controls [15]. To further determine which factor (m.1494C>T or gentamycin) was responsible for this decrease, a Western blot analysis was carried out to examine the levels of four respiratory complex subunits in mutant and control cybrids with tubulin as a loading control. As shown in Figure 1A, the levels of p.MT-CO1 and p.MT-CO2, subunits I and II of cytochrome c oxidase; p.MT-ND5 and p.MT-ND6, subunits 5 and 6 of NADH dehydrogenase were decreased in three mutant cell lines in the absent/present of gentamycin, as compared with those of three control cell lines. According to Figure 1B, the overall level of three mitochondrial translation products in mutant cell lines was decreased dramatically with an average of 39% (*p* = 0.046) except of p.MT-ND6, compared with the mean value in the control cybrids. The average levels of p.MT-CO1, p.MT-CO2, p.MT-ND5 and p.MT-ND6 in mutant cell lines were 65% (*p*<0.001), 50% (*p*<0.001), 68% (*p* = 0.004) and 95% (*p* = 0.24) of the average value in control cybrids respectively. After exposure to gentamycin, no remarkable change in these four proteins’ levels was found among three control cybrids. However, the average levels of p.MT-CO1, p.MT-CO2, p.MT-ND5 and p.MT-ND6 in mutant cell lines became to 66% (*p*<0.001), 41% (*p*<0.001), 50% (*p* = 0.013) and 85% (*p* = 0.09) of those in control cybrids respectively. This result was consistent to previous study that m.1494C>T was responsible for impairment of mitochondrial translation.

### Gentamycin Decreases the Mitochondrial Energy Capacity

**Figure 2:**
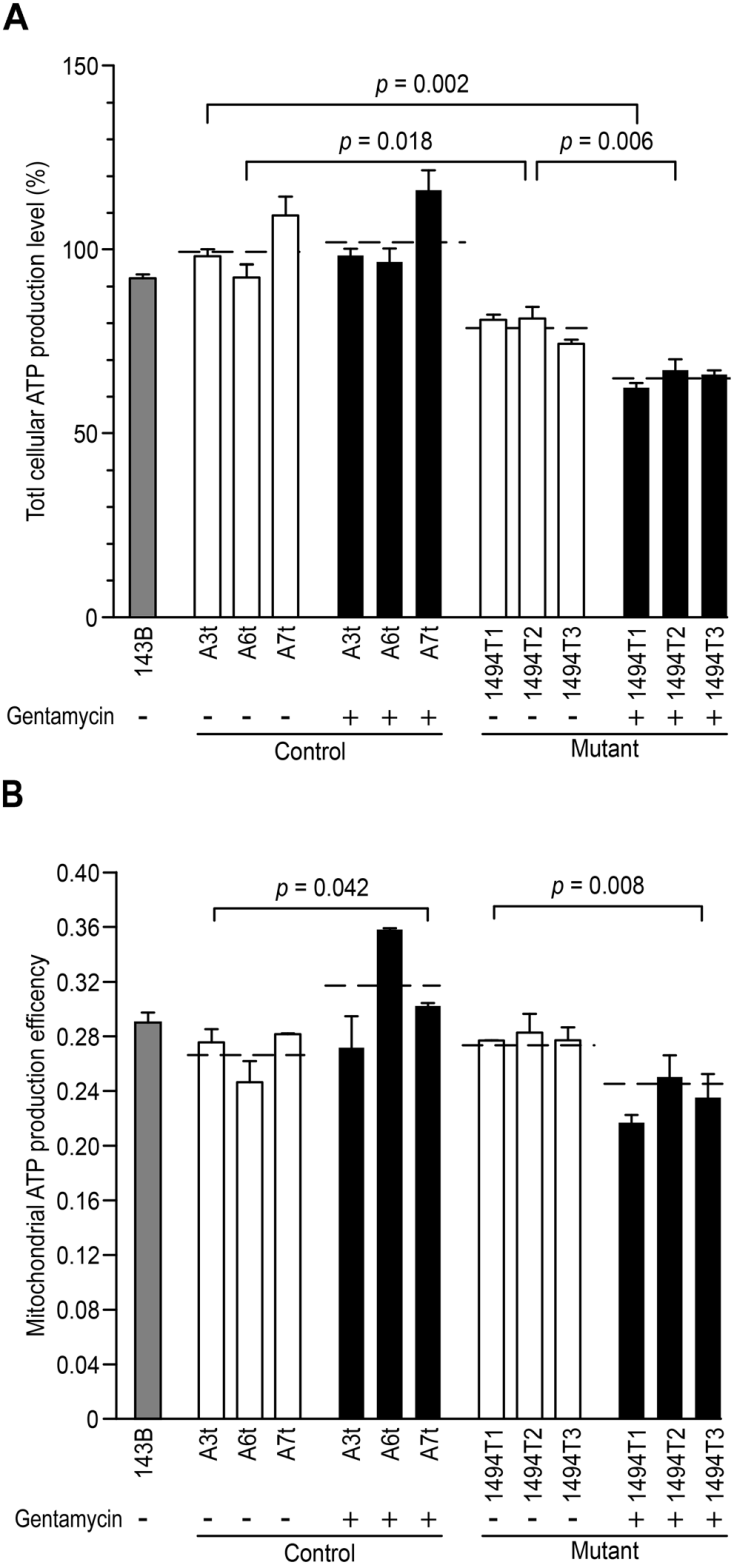
Measurement of cellular ATP production and its efficiency rate. The cells were incubated with 10 mM glucose or 5 mM 2-deoxy-d-glucose plus 5 mM pyruvate to quantify ATP generation through mitochondrial ATP synthesis. The efficiency of ATP production was measured as the mean value of mitochondrial ATP generation *vs.* the total ATP generation in each cell line. (A) ATP level per cell line. (B) Efficiency of ATP production per cell line. At least three independent experiments were performed. Student’s *t* test, between the mutant and control cybrids, without (−) or with (+) gentamycin stimuli.

**Figure 3:**
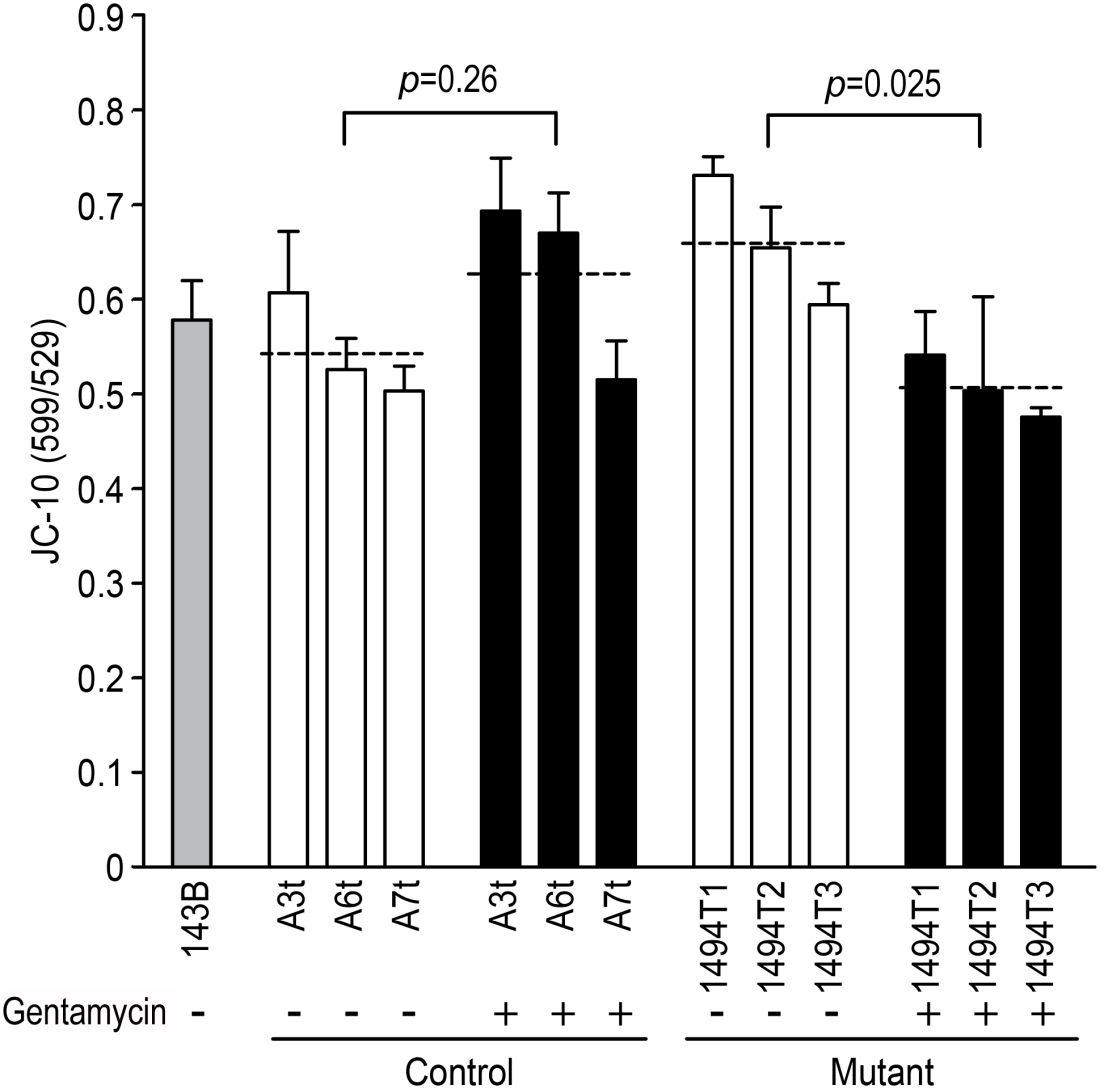
Measurements of the mitochondrial membrane potential. The mitochondrial membrane potential (Δψ_m_) was measured via the JC-10 assay. The ratio of the Ex/Em = 488/590 nm and 488/529 nm fluorescence intensities (FL590/FL529) was recorded to determine the Δψ_m_ level in each sample. The relative ratios of the FL590/FL529 geometric means between the mutant and control cell lines were calculated to reflect Δψ_m_. The data are representative of three independent experiments; Students *t*-test, unpaired, two-tailed.

Given that impaired mitochondrial translation of respiratory chain should decrease the mitochondrial energy capacity, the levels of cellular and mitochondrial ATP in cybrids were detected with or without gentamycin stimulation (Figure 2). In the absent of gentamycin stimulation, 21% (*p* = 0.018) reductions of ATP production was observed in the mutant cybrids, compared with the control cybrids. After exposure to gentamycin, no reduction of ATP production was detected in control cybrids. While 35% (*p* = 0.002) reduction of ATP production was occurred in the double-hit cybrid, compared with the control cybrids. An additional marked decrease of 14% (*p* = 0.006) occurred in double-hit cybrid compared with the mutant cybrids. The efficiency of mitochondrial ATP production was measured as the average value of mitochondrial ATP in the presence of pyruvate and 2-deoxy-D-glucose compared with the average level of cellular ATP per cell line. In the absence of gentamycin, the efficiency ratios for the control and mutant cybrids were similar. After exposure to gentamycin, the efficiency ratio in the control cybrids increased from ∼26% to ∼31% (*p* = 0.042) as a stress response. In contrast, the efficiency ratio in the mutant cybrids decreased significantly from ∼28% to ∼23% (*p* = 0.008). Both the levels of cellular ATP and the efficiency ratio of mitochondrial ATP were inhibited significantly by gentamycin with m.1494C>T together. It was possible that the mitochondrial membrane potential (Δψ_m_) could disturb the energy capacity. We therefore explored Δψ_m_ with gentamycin treatment in cybrids via the JC-10 assay. In the absent of gentamycin, an increase of 20.9% (*p* = 0.09) in Δψ_m_ appeared in mutant cybrid, compared with the control cybrid. In the present of gentamycin, an increase of 14.7% (*p* = 0.26) in Δψ_m_ was detected in the control cybrids (Figure 3). Hence, it appeared that either the m.1494C>T mutation or gentamycin alone has a similar impact on the mitochondrial membrane potential. In contrast, a marked reduction of 31% (*p* = 0.025) was detected in double-hit cybrid compared with the mutant cybrid without gentamycin treatment.

### Gentamycin Elevates Cellular ROS and Decreases Mitochondrial ROS

**Figure 4:**
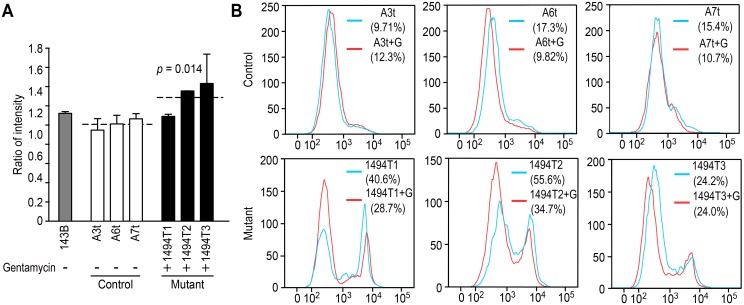
Cellular ROS and mitochondrial ROS production with or without gentamycin. (A) Ratios of the intensity (gentamycin-stimulated vs. non-stimulated) of cellular ROS in 143B.TK^−^ cells (grey column), three control cybrids containing wild-type mtDNA (A3t, A6t & A7t, white columns) and three m.1494C>T cybrids (1494T1/T2/T3, black column). Cells were analyzed with the FC500MCL flow cytometer system (Beckman) using DCFDA (20 µM). (B) Mitochondrial ROS detection using MitoSOX (5 µM) in three control cybrids (A3t, A6t and A7t) and three m.1494C>T cybrids (1494T1/T2/T3), either without (blue) or with (red) gentamycin stimulation. Fluorescence was measured with FACSCalibur (BD Biosciences), and analyzed using FlowJo software.

It was generally accepted that mitochondrial dysfunction should elevate the levels of ROS. Therefore, we investigated whether gentamycin triggered an increase in ROS in either control or mutant cybrids. As shown in Figure 4A, the level of cellular ROS measured with DCFDA in the control cybrids exhibited little change in response to gentamycin. However, in the mutant cybrids, the average ratio of the intensity (gentamycin-stimulated vs. unstimulated) increased markedly to 1.29 (*p* = 0.014), suggesting that gentamycin with m.1494C>T together contributed to increasing cellular ROS levels. Additional, we performed a MitoSOX assay via flow cytometry to determine the levels of mitochondrial ROS (mitoROS) among the cybrids (Figure 4B). As a certain amount of ROS was essential molecules in cellular physiology, the control cybrids without gentamycin stimulation exhibited an average level of 11.7% in mitochondria. No change in mitoROS was detected in the control cells when treated with gentamycin (∼10.5% on average). However, higher mitoROS levels, ranging from 24.2% to 55.6% (∼40% on average), were generated in the mutant cybrid, which further confirms the inherent deficiency of the m.1494C>T mutation concerning mitochondrial dysfunction. Surprisingly, after exposure to gentamycin, the levels of mitoROS in the 1494T1, 1494T2 and 1494T3 cybrids decreased to 28.7%, 34.7% and 24.0% (29.1% on average) respectively.

### Gentamycin Accelerates Changes in Mitochondrial Morphology and Leads to Mitophagy

**Figure 5:**
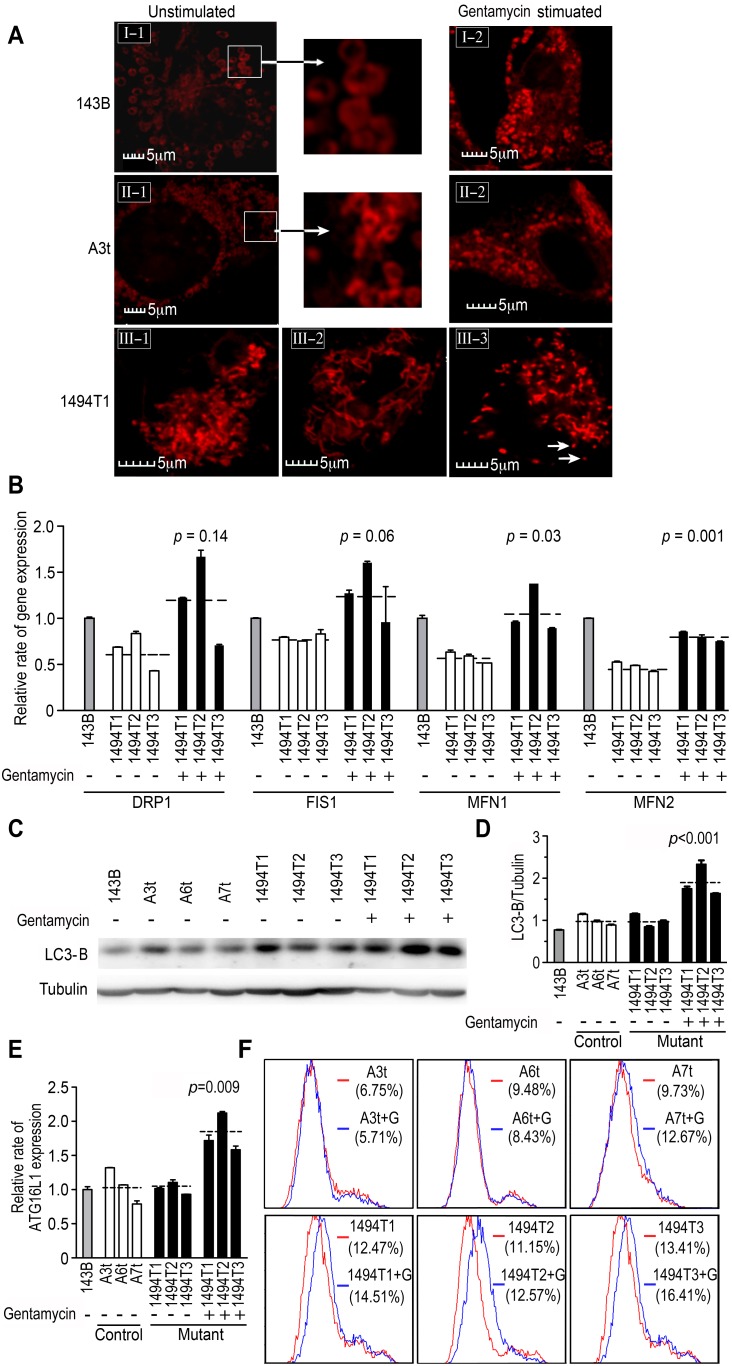
Mitochondrial morphology and mitophagy. (A) Mitochondrial morphology in human 143B.TK^−^ cells (I-1, without gentamycin stimulation; I-2, with gentamycin stimulation), the control A3t cybrid (II-1, without gentamycin stimulation; II-2, with gentamycin stimulation) and the mutant cybrid (III-1, without gentamycin stimulation; III-2 & III-3, with gentamycin stimulation). The mitochondria were visualized using TMRM fluorescence. The two small panels in the middle column show an enlarged view of the boxed regions. The arrowheads indicate fragmented mitochondria. Scale bar: 5 µm. Two independent experiments were conducted. (B) Relative expression rates of genes involved in mitochondrial dynamics in mutant cybrids without (white) *vs.* with (black) gentamycin stimulation. 143B.TK^−^ was used as a normalization control (grey). (C) Western blot analysis of whole-cell extracts to detect LC3-B in 143B.TK^−^ cells, three control cybrids (A3t, A6t and A7t) and three mutant cybrids (1494T1/T2/T3), without (−) or with (+) gentamycin stimulation. Tubulin was probed as a loading control. (D) Quantification of the protein levels (LC3-B/Tubulin) shown in panel C. (E) Relative expression rates of ATG16L1, a marker for the mitophagy process, in the control (white) and mutant cybrids (black) without (−) or with (+) gentamycin stimulation. Data was normalized to the value for 143B.TK^−^ (grey). (F) Analysis of apoptosis in control cybrids (A3t, A6t and A7t) and mutant cybrids (1494T1/T2/T3), without (red) or with (blue) gentamycin stimulation, performed via flow cytometry.

As demonstrated by the above experiments, impairments of mitochondrial protein synthesis, bioenergetic capacity and membrane potential results were induced by the combination of the m.1494C>T mutation and gentamycin. We hypothesize that the mitochondria may be forced to alter their morphology to provide optimal mitochondrial function. Considering the control cybrids (A3t, A6t and A7t) and m.1494C>T cybrids had similar nuclear background as the 143B.TK^−^, TMRM staining was performed in the 143B.TK^−^, A3t and C1494T1 cell lines together with confocal imaging, either with or without gentamycin stimulation. As shown in Figure 5A. An obvious difference in mitochondrial morphology was observed among these cell lines. In 143B and A3t cell lines, the mitochondria were almost uniformly ovoid, as the shape in hepatocyte [27]. In the control cells, little change was detected, even following gentamycin stimulation. However, in C1494T1 cybrids, the mitochondrial morphology was obviously altered, with elongated mitochondria after gentamycin stimulation. As shown in panels III-2 and III-3, most of mitochondria were forced to fuse, showing a tubular appearance and apparently forming extended interconnected mitochondrial networks (III-2) [28]. Smaller fragmented or punctiform mitochondria were also observed in some fused mitochondrial cells (III-3). To determine key molecular components of the mitochondrial fusion/fission machinery that were involved in its dynamics in mutant cybrids, we determined the mRNA expression levels of the key proteins MFN1, MFN2, FIS1, and DRP1 [29]. The mRNA levels of these four proteins were decreased in mutant cybrids compared with the levels in 143B cells. However, the levels of these four proteins increased with gentamycin and m.1494C>T together (Figure 5B), possibly suggesting that with the decrease in energy production, the balance of mitochondrial dynamics was disrupted.

Mitochondrial dynamics can counteract through two activities: first, the rescue of non-functional organelles via fusion (as we observed), and second, the elimination of damaged organelles following fission. Therefore, we monitored the levels of LC3-B, the key mediator of autophagy [30], via western blotting. In the double-hit cybrids, LC3-B accumulated, showing mean 1.9 fold higher level, compared with the control cybrids (*p*<0.001) (Figure 5C and D). This result indicated that mitophagy was sharply stimulated on gentamycin and m.1494C>T stress together. A similar conclusion was drawn from the mRNA levels of ATG16L1 detected in the double-hit cybrids (Figure 5E), which was involved in the formation of autophagosome [31]. Whilst, we conducted an apoptosis assay using flow cytometry to determine whether apoptosis would respond to drug and m.1494C>T stress together. However, in the apoptosis assay (Figure 5F) only mean 8.65% of control cybrids and 12.34% of mutant cybrids respectively underwent apoptosis. After exposure to gentamycin, no change in the level of apoptosis was detected in the control cybrids. The levels of apoptosis in the 1494T1, 1494T2 and 1494T3 cybrids were 14.51%, 12.57% and 16.41% (14.5% on average) respectively.

## Discussion

Deafness can be due to either genetic or environmental causes or a combination of the two. To avoid the implication of nuclear gene, transmitochondrial technology had enabled the study of pathogenic mtDNA mutation at the cellular level. To determine the effect of the m.1494C>T mutation on AG toxicity and deafness, transmitochondrial cybrids were generated as previously described [2], [15]. The nuclear background of the 143B rho0 cell line is notoriously aneuploidy [32]. Therefore, to best address the problem of variance between clones, 6 independent cybird clones derived from three affected matrilineal relative and three genetically unrelated Chinese control subjects were assessed [33]. The m.1555A>G or m.1494C>T was document to responsible for the impairment of mitochondrial translation and subsequent respiration defect [34], [35]. In fact, m.1494C>T mutation markedly reduced the levels of mitochondrial proteins especially in heavy (H) strand (MT-ND5, CO1, CO2) in the present study. As shown in Figure1B, there was no obvious reduction in the level of MT-ND6 in mutant cybrids in the present or absent of gentamycin. It may due to different initiation of mitochondrial transcription in H-strand and L-strand since the transcription from the heavy-strand promoter 2 (HSP2) produces a polycistronic molecule covering the two rRNA genes and 12-mRNA-encoding genes, while transcription from the light-strand promoter (LSP) produces the ND6 mRNA molecule [36], [37]. Similar result was reported in the case of MT-TS1 7511T>C associated with nonsyndromic deafness that significant reduction in the level of MT-ND1 mRNA was found but not MT-ND6 mRNA [38]. Surprisingly, as modifier factor, gentamycin leaded to only an additional 10% decrease in the rate of mitochondrial protein synthesis with m.1494C>T together. Thereby, in this investigation, the significant reduction of mitochondrial protein was contributed to m.1494C>T but not gentamycin. The impairment of mitochondrial translation then resulted in the decrease of ATP synthesis. Obviously, 21% drop of ATP synthesis was respond to m.1494C>T. A much lower drop of ATP (35%) was respond to gentamycin and m.1494C>T together, which was even lower than those in cells carrying MELAS-associated m 3243A>G mutation [39]. Obviously, gentamycin aggravated the defect of mitochondrial energetic capacity. Similarly, under the additional stress of gentamycin in the mutant cybrids, the mitochondrial membrane potential was markedly decreased, which pumped the hydrogen ions across the inner membrane during ATP synthesis. A marked reduction of 31% in Δψ_m_ was at least partly responsible for the dramatic decrease in the ATP capacity. The impairment of both oxidative phosphorylation and mitochondrial membrane potential would elevate the production of ROS in mutant cells. Indeed, the total cellular ROS level was elevated by the stimulus of gentamycin with m.1494C>T together. Interesting, the intensity of mitochondrial ROS in double-hit cybrids (29% on average) was lower than those in m.1494C>T cybrids (40% on average). It was conceivable that some of mitoROS may leaked out the inner membrane when Δψ_m_ was decreased under gentamycin and m.1494C>T stress together.

Furthermore, we found that the manner in which gentamycin accelerated the changes in mitochondrial morphology with the m.1494C>T mutation together. As shown in Figure 5A, the single m.1494C>T was sufficient to force mitochondrial fusion, whereas a single treatment of gentamycin in control cybrid was not. Moreover, gentamycin exposure with m.1494C>T together forced more population of mitochondria to fuse as described elsewhere [40], [41]. The extended interconnected mitochondrial network was suggested to enable efficient mixing of mitochondria to increase respiratory activity and thereby optimize mitochondrial function in an attempt to compensate for the reduction in ATP generation [28]. Likewise, fusion was proposed to represent a pro-survival response to stress and to be crucial for the maintenance of mitochondrial function under stress conditions [42]. It appeared that the rescue of non-functional organelles via fusion in this study was favorable to the balance of mitochondrial dynamics for cell survival. However, the proposed relationship between the mitochondrial networks and the inferred improvement of ATP generation was itself brought into question by our observations, demonstrating that experimentally increasing the extent of such networking was accompanied by a decrease in mitochondrial respiration in double-hit cybrids. This finding was similar to observations made in HeLa cells lacking DRP1 in a previous study [43]. The occurrence of fission was confirmed by the observation of an increased level of mitophagy, which was induced by gentamycin with m.1494C>T together, and supposed to eliminate damaged mitochondria. As predicted by Hickson-Bick [44], the rapid removal of dysfunctional or damaged mitochondria would limit the risk of apoptosis. Accordingly, no considerable change in apoptosis levels was observed, even in the mutant cybrids and double-hit cells, compared with the controls.

Taken together, our observations demonstrate the pathogenic mechanism of gentamycin with m.1494C>T together in hearing loss. As a secondary factor, gentamycin have significant capabilities to enhance the defect in efficiency of ATP production, reduction in the membrane potential and elevation in production of ROS, ultimately leading more mitochondrial to fusion and mitophagy, rather than apoptosis, to counteract the dysfunction in vitro. Our findings strongly suggest that compensatory protection will operate in the early stages of drug-induced hearing loss associated with m.1494C>T mutation to optimize mitochondrial function in a manner that differs from the results of long-term accumulation of drug toxicity [45]. Thus, it may provide valuable information to further understand of mechanistic link between mitochondrial rRNA mutation, AG ototoxicity and hearing loss.

## Conclusion

Aminoglycosides (AGs) as modifying factors, modulated the phenotypic manifestation of m.1555A>G or m.1494C>T mutations and the incomplete penetrance of hearing loss. Our investigation showed that m.1494C>T mutations was responsible for significant reductions in mitochondrial translation and ATP production with an increase in mitochondrial ROS, compared with those of controls. The mitochondrial dysfunction induced by m.1494C>T was aggravated after exposure to gentamycin, via marked additional reduction in ATP production, significant decrease in the mitochondrial membrane potential (Δψm) and more excessive reactive oxygen species (ROS) than those in m.1494C>T cells. Under both gentamycin and m.1494 C>T stress, more dysfunctional mitochondria were forced to fuse and exhibited mitophagy via up-regulated LC3-B, as a compensatory protective response to optimize mitochondrial function, rather than undergo apoptosis. These findings may provide valuable information to further understand of mechanistic link between mitochondrial rRNA mutation, toxicity of AGs and hearing loss.

## Supporting Information

Figure S1 Relative level of mtDNA copy numbers among cybrids. The mtDNA copy number, determined by comparing the ratio of mtDNA to nDNA (18S rRNA) by real-time quantitative PCR, was normalized to the value for 143B.TK^−^. Similar levels of mtDNA copy numbers were found in all cybrids.
(TIF)

Table S1 Primers used for real-time PCR.
(DOCX)
