# Supplementary figures and images for: Aminoglycoside Stress Together with the 12S rRNA 1494C>T Mutation Leads to Mitophagy
Source: PLoS One. 2014 Dec 4;9(12):e114650. doi: 10.1371/journal.pone.0114650 (PMC4256443; doi:10.1371/journal.pone.0114650)

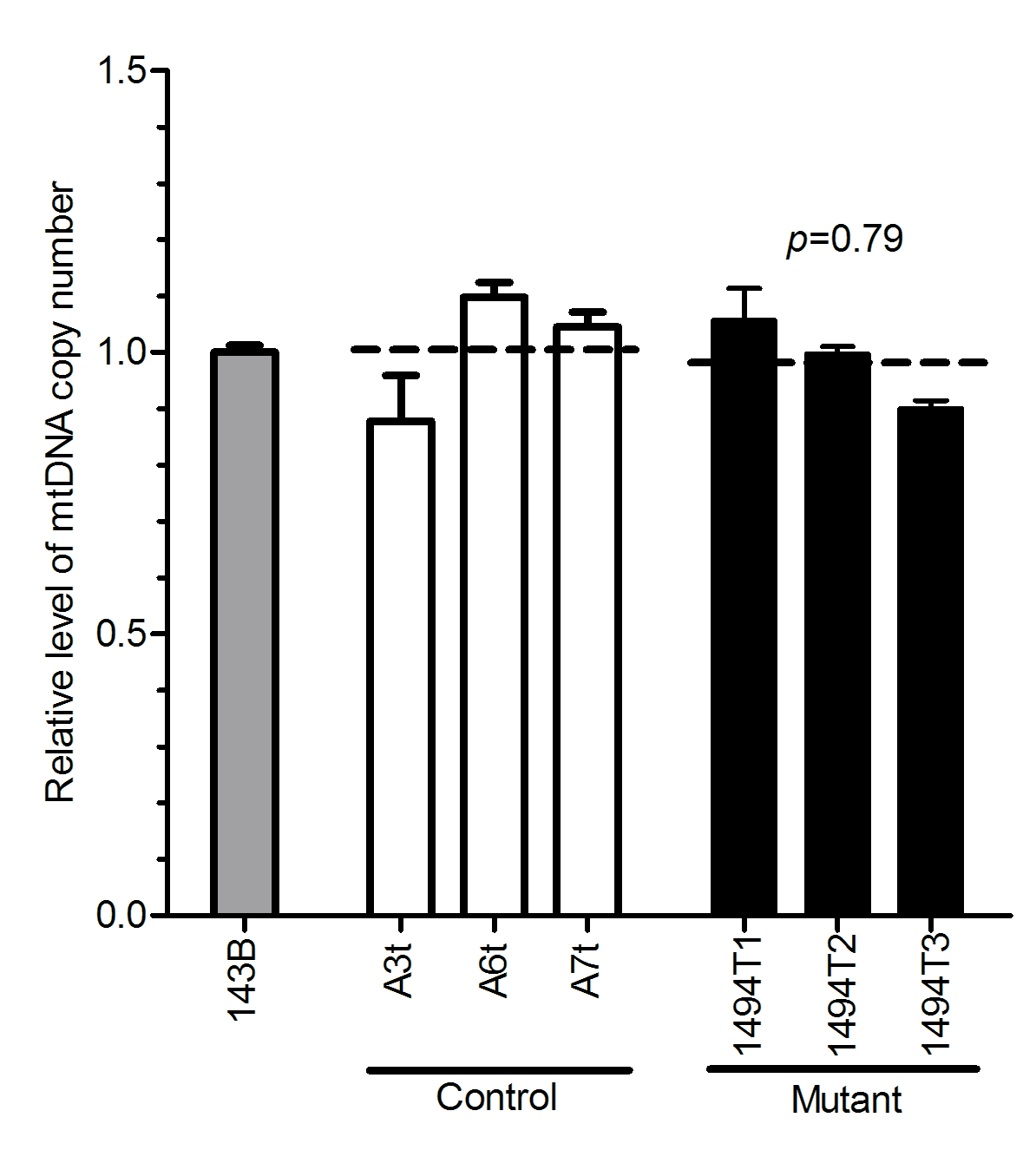

Supplement: Figure S1 — Relative level of mtDNA copy numbers among cybrids. The mtDNA copy number, determined by comparing the ratio of mtDNA to nDNA (18S rRNA) by real-time quantitative PCR, was normalized to the value for 143B.TK−. Similar levels of mtDNA copy numbers were found in all cybrids. (TIF) [file pone.0114650.s001.tif]
