# Supplementary material for: Aminoglycoside Stress Together with the 12S rRNA 1494C>T Mutation Leads to Mitophagy
Source: PLoS One. 2014 Dec 4;9(12):e114650. doi: 10.1371/journal.pone.0114650 (PMC4256443; doi:10.1371/journal.pone.0114650)
Supplement: Table S1 — Primers used for real-time PCR. (DOCX) [file pone.0114650.s002.docx]

**Table S1**. **Primers used for real-time PCR.**

| **GENES** | **PRIMERS: 5' to 3'** |
| --- | --- |
| **MFN1F* | CTCCAGCAACGCCAGATAATGC |
| **MFN1R* | ACTTGTTGGCACAGGCGAGC |
| **MFN2F* | GGATGCTGATGTGTTTGTGCTGG |
| **MFN2R* | AGTCCATGATGAGTCGAACCGC |
| **FIS1F* | CGAGCTGGTGTCTGTGGAGGACC |
| **FIS1R* | TGTCAATGAGCCGCTCCAGTTCC |
| **DRP1F* | AACTTGATCTCATGGATGCGGG |
| **DRP1R* | ATGAACCAGTTCCACACAGCGG |
| *ATG16L1F* | TGTGCGATGCCCTTGGTGCTTTAGT |
| *ATG16L1R* | ACCCAATGAACTAAAACTTTATACG |

*Jendrach M, Mai S, Pohl S, Vöth M, Bereiter-Hahn J (2008) Short- and long-term alterations of mitochondrial morphology, dynamics and mtDNA after transient oxidative stress. Mitochondrion 8: 293-304.
